# Query-Based Multiview Detection for Multiple Visual Sensor Networks

**DOI:** 10.3390/s24154773

**Published:** 2024-07-23

**Authors:** Hung-Min Hsu, Xinyu Yuan, Yun-Yen Chuang, Wei Sun, Ray-I Chang

**Affiliations:** 1 Department of Electrical and Computer Engineering, University of Washington, Seattle, WA 98195, USA; xyuan@uw.edu; 2 Department of Electrical Engineering, National Taiwan University, No. 1, Sec. 4, Roosevelt Road, Taipei 10617, Taiwan; d09921007@ntu.edu.tw; 3 Department of Civil and Environmental Engineering, University of Washington, Seattle, WA 98195, USA; wsun91@uw.edu; 4 Department of Engineering Science and Ocean Engineering, National Taiwan University, No. 1, Sec. 4, Roosevelt Road, Taipei 10617, Taiwan; rayichang@ntu.edu.tw

**Keywords:** multivew detection, query based learning, 2D–3D consistency

## Abstract

In IoT systems, the goal of multiview detection for multiple visual sensor networks is to use multiple camera perspectives to address occlusion challenges with multiview aggregation being a crucial component. In these applications, data from various interconnected cameras are combined to create a detailed ground plane feature. This feature is formed by projecting convolutional feature maps from multiple viewpoints and fusing them using uniform weighting. However, simply aggregating data from all cameras is not ideal due to different levels of occlusion depending on object positions and camera angles. To overcome this, we introduce QMVDet, a new query-based learning multiview detector, which incorporates an innovative camera-aware attention mechanism for aggregating multiview information. This mechanism selects the most reliable information from various camera views, thus minimizing the confusion caused by occlusions. Our method simultaneously utilizes both 2D and 3D data while maintaining 2D–3D multiview consistency to guide the multiview detection network’s training. The proposed approach achieves state-of-the-art accuracy on two leading multiview detection benchmarks, highlighting its effectiveness for IoT-based multiview detection scenarios.

## 1. Introduction

Multiview detection for multiple visual sensor networks is extensively utilized in Internet of Things (IoT) systems to address occlusion issues by integrating multiple camera perspectives. Specifically, IoT systems employing multiview detection leverage synchronized images from various viewpoints, which cover overlapping regions of interest, to compensate for occluded fields of view. Additionally, camera calibration is implemented to aggregate these multiple perspectives onto a ground plane in a bird’s eye view format, thereby alleviating occlusion challenges inherent in monocular view systems.

In IoT systems, the essential component of multiview detection is the aggregation of features from multiple views. The leading method, MVDet [1], utilizes a fully convolutional technique to create feature maps projected onto the ground plane. Convolution is then used to capture neighboring areas across different camera views. However, due to the translation-invariant properties of this convolution-based fusion (where identical computations are applied across views), the resulting feature maps often misalign with the actual locations of objects. Projection from varying camera perspectives introduces diverse distortion patterns. To address these issues, MVDetr [2] leverages deformable attention as an alternative to convolution. Nevertheless, in MVDetr, the equal weighting of each camera during feature map aggregation poses a limitation to its overall effectiveness.

**Figure 1:**
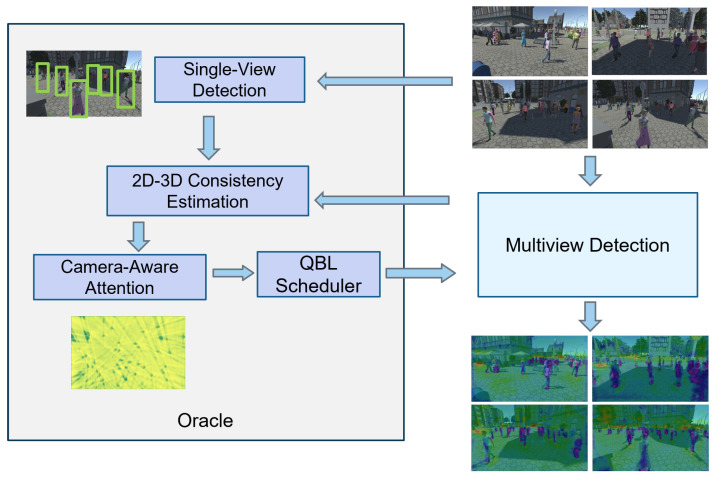
The proposed QMVDet framework.

In this paper, we propose a novel query-based learning solution for the multiview detection task in IoT systems, named QMVDet, which leverages 2D–3D consistency for camera-aware attention via a query-based learning (QBL) scheduler. QBL is a concept in machine learning where a guiding entity known as an oracle directs the learning process [3,4]. We exploit the QBL scheduler to balance the loading of camera-aware attention calculation. Our approach consistently outperforms others, yielding state-of-the-art performance. The proposed method introduces a camera-aware mechanism to enhance multiview detection performance. The holistic pipeline of the proposed solution is shown in Figure 1.

Our contributions with the proposed methods are as follows:

- We propose a new query-based learning solution for the multiview detection task.
- We present a novel camera-aware attention mechanism that utilizes 2D–3D consistency through applying a query-based learning mechanism to aggregate multiview feature maps.
- Our method achieves state-of-the-art performance on both the Wildtrack and MultiviewX benchmarks, which are widely adopted for multiview detection.

The structure of this paper is as follows: Section 2 provides an overview of related work. In Section 3, we present our proposed multiview detection solution, QMVDet. Section 4 details our evaluation of the method using the Wildtrack and MultiviewX benchmarks, including a comparison with current state-of-the-art methods. After that, we discuss the limitations of the proposed method in Section 5. Finally, we conclude the paper in Section 6.

## 2. Related Work

**Multiview Detection.** Multiview detection in pedestrian detection systems effectively mitigates occlusion challenges by using multiple synchronized and calibrated cameras. This technique investigates the correlation between ground plane locations and corresponding bounding boxes across different camera views, enabling a holistic scene description through complementary perspectives. Assuming an average human height in 3D, perspective transformation is employed to estimate 2D bounding boxes in individual camera views. Evaluations of multiview detection systems typically utilize pedestrian occupancy maps on the ground plane [1,5,6]. A pivotal issue in multiview detection is the aggregation of information from multiple views. Methods such as [5] leverage the consistency of neighboring locations for information fusion, while MVDet [1] employs convolution to integrate spatially adjacent locations across views. Despite these advancements, each approach has its drawbacks. For instance, refs. [5,7] necessitate additional neural network architectures; ref. [8] inadequately addresses spatial adjacency; and ref. [1] depends on fixed computations for spatial feature capture. On the other hand, there are some works for multiview tracking [9,10]. Ref. [9] uses Bayesian filter to handle the occlusion, and ref. [10] proposes to integrate the track initialization and re-identification into the Bayesian filter for multiview tracking.

**Transformers.** On the other hand, inspired by the success of transformers [11,12] and their various applications [13,14,15,16,17], researchers have started exploring the use of multi-head self-attention to model relationships between different points. By incorporating positional embeddings, fully connected layers can be used to enhance the model’s ability to handle location sensitivity. Transformers have shown exceptional performance in natural language processing tasks such as machine translation and question answering due to their scalability and capability. In the realm of computer vision, transformer-based models like the image classifier ViT [14] and the object detection model DETR [13] have proven highly effective. To reduce computational complexity, Deformable DETR [18] was introduced, focusing attention only on a few points around a reference, similar to the concept of deformable convolutions [19], making the process fully learnable. In the context of multiview detection, ref. [2] leverages a deformable transformer to create more robust feature maps.

**Multi-Task Learning.** With the advancement of multi-task learning [20,21], end-to-end one-shot multiple object tracking using a single network has gained increasing attention [22,23]. Notably, joint detection and embedding (JDE) [22] and FairMOT [24] have introduced a re-ID branch to the encoder–decoder architecture to train re-identification features and detectors simultaneously for a single camera. The JDE framework significantly reduces inference time by reusing backbone features from the re-ID branch. Traditionally, two-step models for multiple object tracking have outperformed one-step models. However, as highlighted by [24], integrating these two tasks is complex and requires careful handling to prevent failures. In our approach, we employ a single multiview detection network to perform multi-task learning by using perspective transformation to convert 3D detections into 2D detection results.

**Single-Camera Tracking Methods.** Extensive research has been conducted on single-camera tracking (SCT) [25,26,27,28,29], which can be divided into two main categories: tracking by detection and joint detection and embedding (JDE). The superior performance of deep learning-based object detection [30] has led to the dominance of tracking-by-detection methods [25,31,32] in SCT over the past few years. Following the tracking-by-detection approach, JDE emerged as the first method [22,23,33,34,35] to combine object detection and re-identification feature extraction into a single network, thereby accelerating inference time. Track-RCNN [23] enhances Mask RCNN by adding a re-ID head to regress bounding boxes and generate re-ID features for each proposal. In [22], YOLOv3 [36] serves as the base model to enable real-time inference. Generally, the performance of JDE (a two-step method) is lower compared to the tracking-by-detection paradigm (one-shot trackers). In QMVDet, we adopt the detection-by-tracking approach to extract 2D tracking results, which provides reliable 2D detections for consistent 2D–3D estimation.

## 3. Method

Our framework aims to determine the 3D coordinates of each individual using images captured from multiple cameras. With a set of images and their corresponding camera parameters, we seek to detect pedestrian locations within the overlapping fields of view while maintaining 2D–3D consistency constraints. To accomplish this, we introduce a 2D–3D consistency constraint that jointly optimizes the proposed QMVDet and the 2D single-view detection network using consistency and mimic losses. Although QMVDet inherently maintains consistency, the 2D single-view detection network may not always ensure strict 2D–3D consistency, potentially leading to inaccuracies in QMVDet’s results. To address this, we use inconsistent 2D–3D projection results as an attention mechanism to generate distributions based on the inconsistency, weighting the importance of each camera for multiview aggregation. We model learnable latent codes with the conditional probability of 2D–3D detection inconsistency through a 2D–3D consistency estimation.

**Figure 2:**
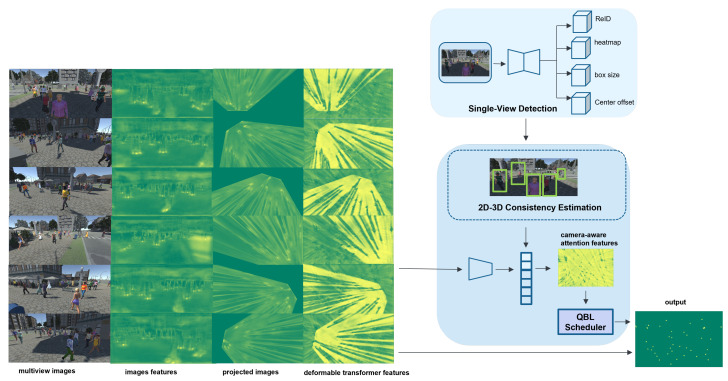
Overview of our QMVDet framework. Initially, the input image is processed through an encoder–decoder network to extract high-resolution feature maps, allowing us to generate 2D MOT results. Subsequently, another encoder extracts image feature maps, which are then projected onto the ground plane. Following this, a deformable transformer is employed to derive feature maps from each camera view. Finally, we utilize camera-aware attention via the QBL scheduler to integrate these deformable transformer-encoded feature maps, creating a final representation for multiview detection.

Next, we will introduce the 2D single-view detection network in Section 3.1, which is followed by a discussion of our QMVDet in Section 3.2. Finally, we will explain how we establish the 2D–3D consistency mechanism based on these two networks in Section 3.3. Figure 2 presents the proposed QMVDet framework with further details provided in Algorithm 1.
**Algorithm 1:** QMVDet Algorithm
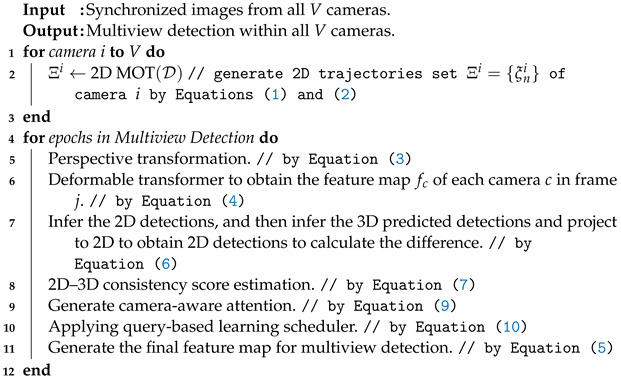


### 3.1. Two-Dimensional (2D) Single-View Detection Network

To evaluate the consistency between 2D and 3D, it is essential to have a 2D single-view detection network. To ensure more accurate 2D detection results, we utilize the detection-by-tracking approach, which leverages tracklet association and interpolation to compensate for missed detections. This is crucial for 2D–3D consistency estimation, as 2D detection performance is often impacted by occlusions present in 2D images. Therefore, we employ a multiple object tracking (MOT) network as our single-view detection component.

Our single-view detection setup follows the configuration of FairMOT [24], utilizing an anchor-free detector. We use DLA-34 [37] to predict heatmaps, object center offsets, and bounding box sizes, and we incorporate 3 × 3 convolutional layers to generate output features. The final layer is a 1 × 1 convolutional layer that produces the final output. The single-view detection branch includes two heads: a heatmap head and a box head. The loss functions are defined as follows:

(1) $$\begin{array}{cc} \; & L_{svHeat}\\ \; & =-\frac{1}{N}\sum_{p}\left\{\begin{array}{cc} {\left(1-{\hat{F}}_{xy}\right)}^{\alpha }log\left({\hat{F}}_{xy}\right) & \mathrm{if}\;F_{xy}=1\\ {\left(1-{\hat{F}}_{xy}\right)}^{\beta }{\left({\hat{F}}_{xy}\right)}^{\alpha }log\left(1-{\hat{F}}_{xy}\right) & \mathrm{otherwise}, \end{array}\right. \end{array}$$

(2) $$L_{box}=\sum_{i=1}^{N}\left|\right|o_{bbox}^{i}-{\hat{o}}_{bbox}^{i}\left|\right|-\lambda_{s}\left|\right|s_{bbox}^{i}-{\hat{s}}_{bbox}^{i}\left|\right|$$

where $\hat{F}$ represents the heatmap, and α and β are the parameters of the focal loss. The heatmap head is responsible for estimating the centers of pedestrians, ideally producing a value of one when it aligns with the ground truth. Conversely, the box offset and size loss functions are employed to enhance the accuracy of pedestrian locations. The single-view detection branch is based on the CenterNet framework [37], which is widely adopted in many anchor-free methods. For each bounding box *i*, $o_{i}^{bbox}$ denotes the corresponding offset, and $s_{i}$ represents its size. The predicted offset and size are denoted by ${\hat{o}}_{i}^{bbox}$ and ${\hat{s}}_{i}^{bbox}$, respectively. $\lambda_{s}$ is a weighting parameter set to 0.1, following the original CenterNet [37].

### 3.2. QMVDet

In this section, we describe the method for leveraging 2D–3D consistency to create an attention mechanism across multiple cameras. We propose a query-based learning framework for multiview detection, wherein the 2D single-view detection network directs the 3D multiview detection network.

A multiview detection system involves two primary steps: projecting feature maps and aggregating multiview data. The first step projects the feature maps from multiple views onto a ground plane (bird’s eye view) through perspective transformation. This is accomplished by extracting feature maps from a 2D single-view detection network and applying perspective transformation [1] to achieve anchor-free representations. This transformation process translates between 2D image pixel coordinates (u,v) and 3D locations (x,y,z). Using the 2D image pixel coordinates, the corresponding 3D world coordinates on the ground plane (where z=0) are calculated to generate the projected feature maps.

(3) $$\begin{array}{cc} \gamma \left(\begin{array}{c} u\\ v\\ 1 \end{array}\right) & =P\left(\begin{array}{c} x\\ y\\ z\\ 1 \end{array}\right)=I\left[R|t\right]\left(\begin{array}{c} x\\ y\\ z\\ 1 \end{array}\right)\\ \; & =\left[\begin{array}{cccc} p_{11} & p_{12} & p_{13} & p_{14}\\ p_{21} & p_{22} & p_{23} & p_{24}\\ p_{31} & p_{32} & p_{33} & p_{34} \end{array}\right]\left(\begin{array}{c} x\\ y\\ z\\ 1 \end{array}\right), \end{array}$$

where γ denotes a scaling factor, and *P* represents the perspective transformation matrix, which is derived from the intrinsic parameter *I* and the extrinsic parameter, consisting of the rotation–translation matrix [R|t].

The second step in multiview detection is the anchor-free aggregation of feature maps. In our framework, we use the encoder from the deformable transformer as our feature extractor to produce aggregated multiview projected feature maps in accordance with the principles of MVDetr [2].

(4) $$\begin{array}{ccc} \; & \; & MVDeformAttn({\left\{f_{\hat{c}}\right\}}_{\hat{c}=1}^{C},p,c)\;\;\;\;\;\;\;\;\;\;\;\;\;\;\;\;\;\;\\ \; & \; & =\sum_{m=1}^{M}W_{m}\sum_{c^{\prime }=1}^{C}\sum_{k=1}^{K}a_{mkc^{\prime }}W_{m}^{\prime }f\left(p+\Delta p_{mkc^{\prime }}\right) \end{array}$$

where *p* denotes the position, and *c* represents the camera ID. $\Delta p_{mkc^{\prime }}$ is the set of position offsets for the deformable reference point, with *k* indicating the number of reference points. $W_{m}$ and $W_{m}^{\prime }$ are the transformations for multi-head *m*.

In this context, treating all camera views with equal weighting for multiview projected feature map aggregation is not optimal due to varying occlusion levels and different visibilities from each camera. Therefore, we propose a query-based learning framework that allows the network to learn attention weights for each camera, enabling adjustable weighted feature map aggregation. This method leverages 2D–3D consistency to guide the learning of the 3D multiview detection network using a 2D single-view detection network.

(5) $$\begin{array}{ccc} \; & \; & QMVDeformAttn({\left\{f_{\hat{c}}\right\}}_{\hat{c}=1}^{C},p,c)\;\;\;\;\;\;\;\;\;\;\;\;\;\;\;\;\;\;\\ \; & \; & =\sum_{m=1}^{M}W_{m}\sum_{c^{\prime }=1}^{C}A_{qbl}\sum_{k=1}^{K}a_{mkc^{\prime }}W_{m}^{\prime }f\left(p+\Delta p_{mkc^{\prime }}\right) \end{array}$$

where $A_{qbl}$ represents the trained camera-aware attention vector based on query-based learning. To determine $A_{qbl}$, we start by inferring the 3D foot point $g_{c,p}$ from the multiview detection network. Using perspective transformation, we convert these to 2D foot point coordinates $f_{3D\to 2D}\left(g_{c,p}\right)$. This allows us to measure the discrepancy $d_{c,p}^{2D}$ between $f_{3D\to 2D}\left(g_{c,p}\right)$ and the 2D foot point coordinates ${\tilde{g}}_{c,j}^{2D}$ predicted by the 2D single-view detection network, as defined in Equation (6). We then calculate the average discrepancy for all pedestrians *p* in Equation (7) for each camera *c*, resulting in $A_{c}$. This serves as the 2D–3D consistency-based camera-aware attention to aid in training the multiview detection network. It is worth noting that projecting 2D detection results into 3D space to compare with predicted 3D coordinates is an alternative method; however, it is less reliable than the 3D to 2D projection approach used in Equation (6).

(6) $$d_{c,p}^{2D}=\underset{j}{\arg\min}{∥\left({\tilde{g}}_{c,j}^{2D}-f_{3D\to 2D}\left(g_{c,p}\right)\right)∥}_{2}$$

(7) $$A_{c}=\frac{1}{P}\sum_{p}^{P}d_{c,p}$$

Within the camera-aware attention model, we apply attention-weighted averaging to a sequence of image features. The concept of camera-aware attention for aggregating multiview data is defined as follows:

(8) $$F=\frac{1}{C}\sum_{c=1}^{C}A_{c}f_{c}.$$

The network responsible for generating attention processes a sequence of image-level deformable transformer features $f_{c}$ and produces *C* attention scores. Our attention mechanism involves two key steps: “spatial convolution” and “cross-camera convolution”. First, a spatial convolution layer is applied to each frame from every camera, resulting in a *d*-dimensional feature for each frame. Next, a cross-camera convolution layer combines these frame-level features from all cameras to create temporal attentions $\xi_{c}$. The attention scores $A_{c}$ are then multiplied by $\xi_{c}$, and a softmax function is applied to produce the final camera-aware attention vector $A_{c}$.

(9) $$A_{c}=\frac{A_{c}e^{\xi_{c}}}{\sum_{c=1}^{C}A_{c}e^{\xi_{c}}}$$

**Figure 3:**
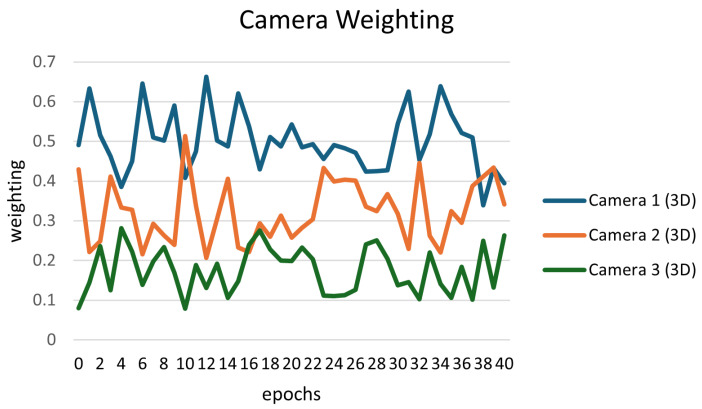
An example of 2D–3D consistent restricted camera-aware weighting for three cameras of the Wildtrack dataset. The weight order changes multiple times during the training; in this case, the QBL scheduler activates at epochs 11, 12, and 39.

Due to the computational intensity involved in the 2D–3D consistency estimation, substantial computational resources are needed. Therefore, we introduced a query-based learning (QBL) scheduler to manage the frequency of guiding the multiview detection learning process. When there are significant changes in the distribution of the camera-aware attention vector, meaning the relative weights of the cameras shift, the QBL scheduler adjusts by providing the camera-aware attention vector to generate the final attention vector $A_{qbl}$ to steer the learning of multiview detection. Ultimately, a pedestrian occupancy map is employed to generate the multiview detection results via ground plane convolution. In Figure 3, we illustrate how to use weight order to monitor the changes in the distribution of the camera-aware attention vector. If the weight order changes, the QBL scheduler will be activated immediately.

(10) $$A_{qbl}=\left\{\begin{array}{cc} A_{c} & \mathrm{if}\;\mathrm{QBL}\;\mathrm{Scheduler}\;\mathrm{activated}\;\text{i.e.},\;(1-{\mathrm{Entropy}}_{t}/{\mathrm{Entropy}}_{t-1})>0.1,\\ 1 & \mathrm{otherwise}. \end{array}\right.$$

### 3.3. Training Scheme

Multiview detection essentially involves detecting key points with the objective of multiview systems being to estimate pedestrian occupancy on the ground plane [1,6]. We employ a heatmap regression method to predict the likelihood of pedestrian occupancy, which is inspired by the approach used in CornerNet [38]. In the QMVDet framework, we also train a single-view detection network to produce 2D detection results. These results are then used for 2D–3D consistency estimation, which in turn guides the training of the camera-aware attention mechanism.

**Training Scheme for Single-view Detection.** We train the 2D MOT by combining multiple loss functions, including the re-identification (ReID) loss in our single-view detection branch. This is necessary to use tracklet association for obtaining reliable 2D detections for 2D–3D consistency estimation. Drawing inspiration from uncertainty losses, we automatically balance these losses using Equation (11).

(11) $$\begin{array}{cc} L_{2DMOT}= & \frac{1}{3}(\frac{1}{e^{w1}}\left(L_{svHeat}+L_{box}\right)+\frac{1}{e^{w2}}L_{ReID}\\ \; & +\frac{1}{e^{w3}}L_{cam_{cls}}+w_{1}+w_{2}+w_{3}). \end{array}$$

where $w_{1}$, $w_{2}$, and $w_{3}$ are learnable parameters. Our loss for 2D MOT is inspired by FariMOT [24], and the re-identification (ReID) loss $L_{ReID}$ is cross-entropy loss. *N* denotes the total number of samples. *K* denotes the total number of classes. $L_{i}\left(k\right)$ represents the actual distribution (typically a one-hot encoded vector) for the *i*-th sample in the *K*-th class. p(k) represents the predicted probability of the *K*-th class by the model. For $L_{cam_{cls}}$, we use another cross-entropy loss to learn the camera classification. *C* means the number of cameras.

(12) $$L_{ReID}=-\sum_{i=1}^{N}\sum_{k=1}^{K}L_{i}\left(k\right)\log\left(p\left(k\right)\right)$$

(13) $$L_{cam_{cls}}=-\sum_{i=1}^{N}\sum_{c=1}^{C}L_{i}\left(c\right)\log\left(p\left(c\right)\right)$$

**Training Scheme of Multiview Detection.** The goal of the multiview detection network is to generate a heatmap that represents the pedestrian occupancy likelihood score ${\hat{s}}_{p}$ for each position *p* on the ground plane. Inspired by the focal loss [39] and using a Gaussian-smoothed target *s*, the loss function for multiview detection can be formulated as follows:

(14) $$\begin{array}{cc} \; & L_{mvHeat}\\ \; & =\frac{1}{N}\sum_{xy}\left\{\begin{array}{cc} {\left(1-{\hat{s}}_{p}\right)}^{\alpha }log\left({\hat{s}}_{p}\right)) & \mathrm{if}\;s_{p}\;=\;1\\ {\left(1-s_{p}\right)}^{\beta }{\left({\hat{s}}_{p}\right)}^{\alpha }log\left(1-{\hat{s}}_{p}\right) & \mathrm{otherwise}, \end{array}\right. \end{array}$$

where *N* represents the total number of pedestrians on the ground plane and $s_{p}$ indicates the ground truth position of the target *s*. Similar to the approach in MVDetr [2], we also predict an offset to account for the lower resolution of the output heatmap compared to the ground truth, allowing us to adjust for the missing decimal precision.

(15) $$L_{off}=\frac{1}{N}\sum_{p|s_{p}^{\varepsilon_{i}}=1}\left|\delta_{p}-\left(\frac{p}{r}-\left\lfloor \frac{p}{r}\right\rfloor \right)\right|$$

where $\delta_{p}$ represents the positional offset and *r* is the downsampling parameter. Additionally, we incorporate a bounding box regression loss based on the L1 distance into our final loss function. Hence, the complete loss function is as follows:

(16) $$\begin{array}{cc} \; & L_{3DMVN}=L_{mvHeat}+L_{off}\\ \; & +\frac{1}{C}\sum_{c}\left(L_{svHeat,c}+L_{off,c}+0.1\times L_{box,c}\right). \end{array}$$

$L_{svHeat,c}$, $L_{off,c}$ and $L_{box,c}$ represent the image-level loss for a specific camera *c*.

## 4. Experiments

In this section, we evaluate the performance of our proposed QMVDet method using the Wildtrack and MultiviewX datasets, which are key benchmarks for multiview detection. We also compare the results of our method with those of the current leading multiview detection approaches.

### 4.1. Experiment Settings

#### 4.1.1. Dataset

Wildtrack [6] is a real-world dataset that focuses on a 12 × 36 square meter area, which is covered by seven synchronized cameras. The ground plane, measuring 12 m by 36 m, is divided into a grid with a resolution of 480 × 1440, where each cell is 2.5 cm by 2.5 cm. This dataset consists of 400 frames with a resolution of 1080 × 1920 pixels. The first 360 frames are used for training, while the remaining 40 frames are reserved for testing. On average, each frame contains 20 people, and each location is viewed by approximately 3.74 cameras.

Another multiview detection dataset, MultiviewX [1] is a synthetic dataset generated using the Unity engine and human models from PersonX [40]. It captures a 16 × 25 square meter city square using 6 cameras. The ground plane is divided into a 640 × 1000 grid with images at a resolution of 1080 × 1920 pixels. Similar to Wildtrack, it includes 400 frames, with the last 40 frames designated for evaluation. Each frame typically contains 40 people, and each location is covered by an average of 4.41 cameras.

#### 4.1.2. Metrics

For multiview detection tasks, the commonly used metrics are Multiple Object Detection Accuracy (MODA), Multiple Object Detection Precision (MODP), precision, and recall. MODA, which accounts for both false positives and false negatives, is the primary metric for evaluating performance. Unlike monocular-view detection systems that assess estimated bounding boxes, multiview detection systems evaluate the estimated ground plane occupancy map. Therefore, the distance between the detected pedestrian location and the corresponding ground truth is measured with a threshold of 0.5 m to classify true positives [1,2].

#### 4.1.3. Implementation Details

Building on the approach of FairMOT [24], we employ a modified version of DLA-34 [37] as our backbone. The initial learning rate is set to 1 × 10^−4^, which decays to 1 × 10^−5^ after 20 epochs. We use a batch size of 12 and the Adam optimizer for training. For the multiview detection component, we adopt ResNet18 as our feature extractor, following the methodology of MVDetr [2]. The world grid is downsampled by a factor of γ=4. The deformable multiview transformer consists of 3 encoder layers, which each have 8 heads and 4 reference points. The Adam optimizer is used with a learning rate of 5 × 10^−4^. Our multiview detection implementation is based on MVDeTr [1,2]; thus, the input images are downsampled to a resolution of 720 × 1280, producing output features of size 90 × 160. All experiments are conducted on an Nvidia A6000 GPU (depending on the framework) with a batch size of 1.

### 4.2. Evaluation of QMVDet

**Figure 4:**
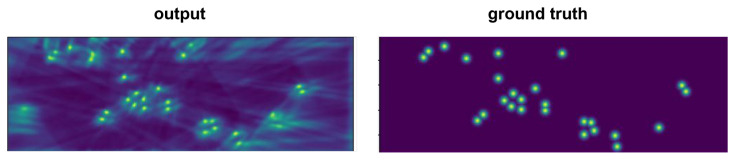
The output heatmap of Wildtrack.

**Figure 5:**
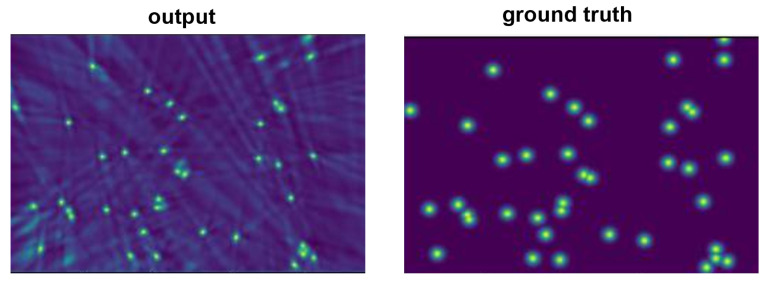
The output heatmap of MultiviewX.

**Figure 6:**
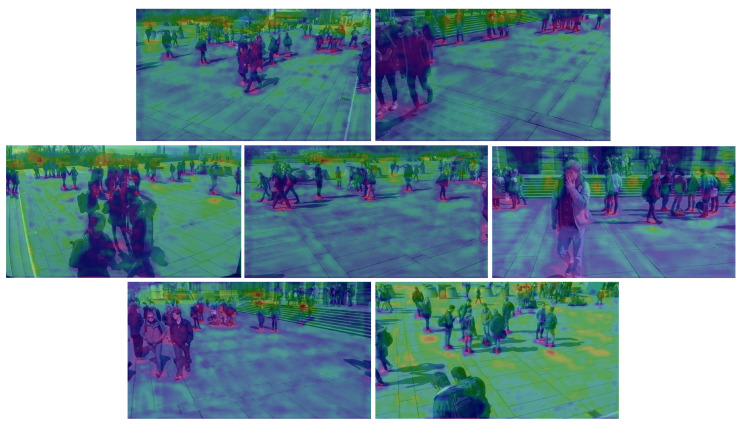
Qualitative results for detected 2D foot points of QMVDet on Wildtrack.

**Figure 7:**
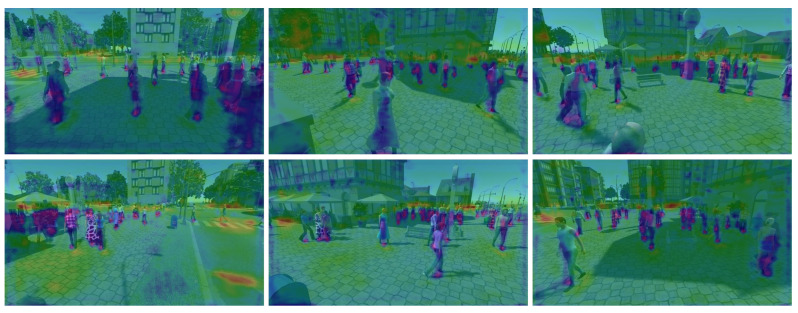
Qualitative results for detected 2D foot points of QMVDet on MultiviewX.

We evaluated our proposed method against state-of-the-art multiview detectors using the Wildtrack and MultiviewX datasets. As shown in Table 1, QMVDet achieved a 1.6% increase in MODA on the Wildtrack dataset, reaching 93.1%, compared to MVDetr [2]. For Wildtrack, our model improved both the MODA and recall metrics with a slight increase in MODP by 0.5% and an overall recall improvement of approximately 2.4%. Similarly, our method either matched or outperformed MVDetr across all four metrics on the MultiviewX dataset. In Table 2, QMVDet is shown to have achieved an MODA of 95.1% on the MultiviewX dataset, which is a 1.4% enhancement. Figure 4 and Figure 5 illustrate the heatmap results for QMVDet, while Figure 6 and Figure 7 present quantitative results. These heatmaps show that QMVDet’s occupancy probabilities closely align with the ground truth, highlighting the effectiveness of the camera-aware attention-based aggregation. For these figures, brighter colors indicate higher probabilities for detected objects, while darker or cooler colors indicate lower probabilities for detected objects.

### 4.3. Ablation Studies

In this section, we present ablation studies focusing on camera selection, different portions of training data, the complexity of QMVDet, enhancements with various convolution types, and improvements with different methods for attention vector generation.

**Camera Selection.** Table 3 illustrates that the algorithm remains functional even if one or more cameras fail during operation. Experimental results indicate that selecting five cameras yields the optimal performance. This insight underpins the motivation for training the camera-aware attention vector to achieve the best results.

**Different Portions of Training Data.** We conducted an ablation study to examine the algorithm’s performance with varying portions of the training data (e.g., 90%, 80%, 70%, …, 10% of the datasets), as shown in Table 4.

**Complexity of QMVDet.** The complexity of QMVDet, as detailed in Table 5, is significantly greater than that of MVDetr, both in terms of computational complexity and the number of parameters. Consequently, the QBL scheduler is essential to enhance training efficiency. However, Table 1 and Table 2 indicate that better performance can be achieved even without the QBL scheduler.

**Enhancements with Various Convolution Types.** We modified the camera-aware attention mechanism by incorporating different types of convolution and evaluated the performance, as presented in Table 6. The experimental results indicate that using camera-aware attention with the deformable transformer achieves the best performance, surpassing other convolution methods by over 1.7% in MODA. The performance gap between applying camera-aware attention to deformable convolution and the transformer is relatively small.

**Improvements with Different Methods for Attention Vector Generation.** Table 7 compares the effectiveness of using softmax and sigmoid functions for generating attention vectors. The experimental findings demonstrate that the softmax function is more effective for the camera-aware attention mechanism.

## 5. Limitations

The proposed method necessitates a consistent camera configuration between the training and testing datasets. Experimental results of camera selection indicate that the failure of certain cameras does not impede the normal operation of the proposed method. Furthermore, QMVDet is specifically designed for the multiview detection task, and real-time application is beyond its current scope. It is important to note that the method does not facilitate the detection of 3D bounding boxes due to the unknown height of the objects. Additionally, incorporating more cameras will inevitably increase the training and inference time required.

## 6. Conclusions

In this paper, we explore the integration of 2D views to guide learning in multiview detection through a query-based learning approach. We observe that assigning equal weights to each view is ineffective for multiview feature aggregation across multiple cameras due to varying object movement patterns. To address this, we introduce QMVDet, which is a novel multiview detection method guided by a 2D single-view detection network. QMVDet utilizes a new camera-aware attention mechanism designed to weigh the significance of each camera, enabling the fusion of feature maps from different positions across multiple views via QBL scheduler. Additionally, we propose a 2D–3D consistency score that maintains multiview 2D–3D consistency during feature aggregation. Our approach sets a new benchmark for performance on the Wildtrack and MultiviewX multiview detection datasets.

## Figures and Tables

**Table 1:** Results comparison on Wildtrack dataset.

Method	MODA	MODP	Precision	Recall
RCNN and clustering [41]	0.113	0.184	0.68	0.43
PMO-CNN [7]	0.232	0.305	0.75	0.55
DeepMCD [8]	0.678	0.642	0.85	0.82
Deep-Occlusion [5]	0.741	0.538	0.95	0.80
MVDet [1]	0.882	0.757	0.947	0.936
MVDetr [2]	0.915	0.821	**0.974**	0.940
3DROM [42]	**0.935**	0.759	0.972	0.962
**Ours (w/o scheduler)**	**0.935**	0.818	**0.974**	0.960
**Ours**	0.931	**0.826**	0.966	**0.964**

**Table 2:** Results comparison on MultiviewX dataset.

Method	MODA	MODP	Precision	Recall
RCNN & clustering [41]	0.187	0.464	0.635	0.439
DeepMCD [8]	0.700	0.730	0.857	0.833
Deep-Occlusion [5]	0.752	0.547	0.978	0.802
MVDet [1]	0.839	0.796	0.968	0.867
MVDetr [2]	0.937	0.913	0.995	0.942
3DROM [42]	0.950	0.849	0.990	**0.961**
**Ours (w/o scheduler)**	**0.953**	**0.927**	0.994	0.959
**Ours**	0.951	0.922	**0.996**	0.955

**Table 3:** Results comparison by using 2D–3D consistency to select cameras for multiview detection network as input on Wildtrack dataset. c1 means to select the camera with the highest 2D–3D consistency; c12 means to select camera 1 and camera 2, and so on.

Method	MODA	MODP	Precision	Recall
c1	0.779	0.769	0.973	0.801
c12	0.873	0.802	0.970	0.901
c123	0.898	0.813	0.976	0.921
c1234	0.919	0.814	**0.977**	0.941
c12345	**0.934**	0.817	**0.977**	0.956
c123456	0.913	0.816	0.969	0.943
all	0.931	**0.826**	0.966	**0.964**

**Table 4:** Results of different portions of training data on Wildtrack.

Training Ratio (%)	MODA	MODP	Precision	Recall
10	0.642	0.767	0.974	0.660
20	0.744	0.774	0.973	0.765
30	0.798	0.792	0.974	0.820
40	0.797	0.799	0.970	0.823
50	0.831	0.812	0.966	0.861
60	0.863	0.814	0.960	0.900
70	0.906	0.827	0.968	0.937
80	0.927	0.827	0.979	0.948
90	0.931	0.826	0.966	0.964

**Table 5:** The FLOPs of the proposed method.

	QMVDet	MVDetr
Total params	28,816,047	16,537,703
Total FLOPs	603,071,144,960	530,816,716,800

**Table 6:** Improvements over different convolution on Wildtrack dataset.

Method	MODA	MODP	Precision	Recall
convolution	0.895	0.817	0.972	0.921
deformable convolution	0.912	0.825	**0.977**	0.934
transformer	0.914	0.823	0.968	0.945
deformable transformer	**0.931**	**0.826**	0.966	**0.964**

**Table 7:** Results comparison of different attention vector generation on Wildtrack dataset.

Method	MODA	MODP	Precision	Recall
softmax	**0.931**	**0.826**	**0.966**	**0.964**
sigmoid	0.922	0.818	0.960	0.962

## Data Availability

The data that support the findings of this study are available from the corresponding author upon reasonable request.

## References

[1] Hou Y., Zheng L., Gould S. Multiview Detection with Feature Perspective Transformation. Proceedings of the Computer Vision–ECCV 2020: 16th European Conference.

[2] Hou Y., Zheng L. Multiview detection with shadow transformer (and view-coherent data augmentation). Proceedings of the 29th ACM International Conference on Multimedia.

[3] Hsu H.-M., Chang R.-I., Ho J.-M. (2017). Query-based-learning genetic algorithm to construct mobile-oriented catalogs in m-commerce. IEEE Access.

[4] Chang R.-I., Hsu H.-M., Lin S.-Y., Chang C.-C., Ho J.-M. (2017). Query-based learning for dynamic particle swarm optimization. IEEE Access.

[5] Baqué P., Fleuret F., Fua P. Deep occlusion reasoning for multi-camera multi-target detection. Proceedings of the IEEE International Conference on Computer Vision.

[6] Chavdarova T., Baqué P., Bouquet S., Maksai A., Jose C., Bagautdinov T., Lettry L., Fua P., Van Gool L., Fleuret F. Wildtrack: A multi-camera hd dataset for dense unscripted pedestrian detection. Proceedings of the IEEE Conference on Computer Vision and Pattern Recognition.

[7] Fleuret F., Berclaz J., Lengagne R., Fua P. (2007). Multicamera people tracking with a probabilistic occupancy map. IEEE Trans. Pattern Anal. Mach. Intell..

[8] Chavdarova T., Fleuret F. (2017). Deep multi-camera people detection. Proceedings of the 2017 16th IEEE International Conference on Machine Learning and Applications (ICMLA).

[9] Ong J., Vo B.T., Vo B.N., Kim D.Y., Nordholm S. (2020). A Bayesian filter for multi-view 3D multi-object tracking with occlusion handling. IEEE Trans. Pattern Anal. Mach. Intell..

[10] Van Ma L., Nguyen T.T.D., Vo B.N., Jang H., Jeon M. (2024). Track initialization and re-identification for 3D multi-view multi-object tracking. Inf. Fusion.

[11] Devlin J., Chang M.W., Lee K., Toutanova K. (2018). Bert: Pre-training of deep bidirectional transformers for language understanding. arXiv.

[12] Vaswani A., Shazeer N., Parmar N., Uszkoreit J., Jones L., Gomez A.N., Kaiser Ł., Polosukhin I. (2017). Attention is all you need. Advances in Neural Information Processing Systems 30.

[13] Carion N., Massa F., Synnaeve G., Usunier N., Kirillov A., Zagoruyko S. (2020). End-to-end object detection with transformers. Proceedings of the European Conference on Computer Vision.

[14] Dosovitskiy A., Beyer L., Kolesnikov A., Weissenborn D., Zhai X., Unterthiner T., Dehghani M., Minderer M., Heigold G., Gelly S. (2020). An image is worth 16×16 words: Transformers for image recognition at scale. arXiv.

[15] Sun C., Myers A., Vondrick C., Murphy K., Schmid C. Videobert: A joint model for video and language representation learning. Proceedings of the IEEE/CVF International Conference on Computer Vision.

[16] Yang F., Yang H., Fu J., Lu H., Guo B. Learning texture transformer network for image super-resolution. Proceedings of the IEEE/CVF Conference on Computer Vision and Pattern Recognition.

[17] Ye L., Rochan M., Liu Z., Wang Y. Cross-modal self-attention network for referring image segmentation. Proceedings of the IEEE/CVF Conference on Computer Vision and Pattern Recognition.

[18] Zhu X., Su W., Lu L., Li B., Wang X., Dai J. (2020). Deformable detr: Deformable transformers for end-to-end object detection. arXiv.

[19] Dai J., Qi H., Xiong Y., Li Y., Zhang G., Hu H., Wei Y. Deformable convolutional networks. Proceedings of the IEEE International Conference on Computer Vision.

[20] Kokkinos I. Ubernet: Training a universal convolutional neural network for low-, mid-, and high-level vision using diverse datasets and limited memory. Proceedings of the IEEE Conference on Computer Vision and Pattern Recognition.

[21] Chen Z., Badrinarayanan V., Lee C.Y., Rabinovich A. (2018). Gradnorm: Gradient normalization for adaptive loss balancing in deep multitask networks. Proceedings of the International Conference on Machine Learning.

[22] Wang Z., Zheng L., Liu Y., Li Y., Wang S. (2020). Towards real-time multi-object tracking. Proceedings of the European Conference on Computer Vision.

[23] Voigtlaender P., Krause M., Osep A., Luiten J., Sekar B.B.G., Geiger A., Leibe B. Mots: Multi-object tracking and segmentation. Proceedings of the IEEE/CVF Conference on Computer Vision and Pattern Recognition.

[24] Zhang Y., Wang C., Wang X., Zeng W., Liu W. (2021). Fairmot: On the fairness of detection and re-identification in multiple object tracking. Int. J. Comput. Vis..

[25] Tang S., Andriluka M., Andres B., Schiele B. Multiple People Tracking by Lifted Multicut and Person Re-identification. Proceedings of the IEEE Conference on Computer Vision and Pattern Recognition.

[26] Wang G., Wang Y., Zhang H., Gu R., Hwang J.N. Exploit the Connectivity: Multi-object Tracking with TrackletNet. Proceedings of the 27th ACM International Conference on Multimedia.

[27] Tang Z., Wang G., Xiao H., Zheng A., Hwang J.N. Single-camera and Inter-camera Vehicle Tracking and 3D Speed Estimation Based on Fusion of Visual and Semantic Features. Proceedings of the IEEE Conference on Computer Vision and Pattern Recognition Workshops.

[28] Cai J., Wang Y., Zhang H., Hsu H.M., Ma C., Hwang J.N. (2020). IA-MOT: Instance-Aware Multi-Object Tracking with Motion Consistency. arXiv.

[29] Zhang H., Wang Y., Cai J., Hsu H.M., Ji H., Hwang J.N. LIFTS: Lidar and Monocular Image Fusion for Multi-Object Tracking and Segmentation. Proceedings of the BMTT Challenge Workshop, IEEE Conference on Computer Vision and Pattern Recognition.

[30] Liu S., Huang D., Wang Y. Adaptive nms: Refining pedestrian detection in a crowd. Proceedings of the IEEE Conference on Computer Vision and Pattern Recognition.

[31] Liu C., Yao R., Rezatofighi S.H., Reid I., Shi Q. (2019). Model-free tracker for multiple objects using joint appearance and motion inference. IEEE Trans. Image Process..

[32] Zhu J., Yang H., Liu N., Kim M., Zhang W., Yang M.H. Online multi-object tracking with dual matching attention networks. Proceedings of the European Conference on Computer Vision (ECCV).

[33] Liang C., Zhang Z., Zhou X., Li B., Zhu S., Hu W. (2020). Rethinking the competition between detection and reid in multi-object tracking. arXiv.

[34] Pang J., Qiu L., Li X., Chen H., Li Q., Darrell T., Yu F. Quasi-dense similarity learning for multiple object tracking. Proceedings of the IEEE/CVF Conference on Computer Vision and Pattern Recognition.

[35] Lu Z., Rathod V., Votel R., Huang J. Retinatrack: Online single stage joint detection and tracking. Proceedings of the IEEE/CVF Conference on Computer Vision and Pattern Recognition.

[36] Redmon J., Farhadi A. (2018). Yolov3: An incremental improvement. arXiv.

[37] Zhou X., Wang D. (2019). Objects as points. arXiv.

[38] Law H., Deng J. Cornernet: Detecting objects as paired keypoints. Proceedings of the European Conference on Computer Vision (ECCV).

[39] Lin T.Y., Goyal P., Girshick R., He K., Dollár P. Focal loss for dense object detection. Proceedings of the IEEE International Conference on Computer Vision.

[40] Sun X., Zheng L. Dissecting person re-identification from the viewpoint of viewpoint. Proceedings of the IEEE/CVF Conference on Computer Vision and Pattern Recognition.

[41] Xu Y., Liu X., Liu Y., Zhu S.C. Multi-view people tracking via hierarchical trajectory composition. Proceedings of the IEEE Conference on Computer Vision and Pattern Recognition.

[42] Qiu R., Xu M., Yan Y., Smith J.S., Yang X. (2022). 3D Random Occlusion and Multi-layer Projection for Deep Multi-camera Pedestrian Localization. Proceedings of the Computer Vision–ECCV 2022: 17th European Conference.

